# The role of atorvastatin in suppressing tumor growth of uterine fibroids

**DOI:** 10.1186/s12967-018-1430-x

**Published:** 2018-03-09

**Authors:** Zhaojun Shen, Saisai Li, Bo Sheng, Qi Shen, Lu-Zhe Sun, Haiyan Zhu, Xueqiong Zhu

**Affiliations:** 1 Department of Obstetrics and Gynecology, The Second Affiliated Hospital of Wenzhou Medical University, No. 109 Xueyuan Xi Road, Wenzhou, 325027 Zhejiang China; 2 Departments of Cell Systems & Anatomy, School of Medicine, University of Texas Health Science Center at San Antonio, San Antonio, TX USA

**Keywords:** Uterine fibroids, Atorvastatin, HMG-CoA, MAPK

## Abstract

**Background:**

Medical therapeutic options remain quite limited for uterine fibroids treatment. Statins, competitive inhibitors of 3-hydroxy-3-methylglutaryl-coenzyme A reductase, have anti-tumoral effects on multiple cancer types, however, little is known about their effects on uterine fibroids.

**Methods:**

Initially, we conducted a retrospective study of 120 patients with uterine fibroids and hyperlipidemia from the Second Affiliated Hospital of Wenzhou Medical University. Then, we evaluated the effect of atorvastatin on proliferation and apoptosis both in immortalized uterine fibroids cells and primary uterine fibroids cells. Furthermore, the molecular mechanism by which atorvastatin suppressed uterine fibroids cell growth was explored.

**Results:**

Our results showed that atorvastatin use for 1 or 2 years significantly suppressed growth of uterine fibroids. Atorvastatin inhibited the proliferation of immortalized and primary uterine fibroids cells in a dose and time-dependent manner and stimulated apoptosis of uterine fibroids cells by inducing caspase-3 activation, up-regulating Bim and down-regulating Bcl-2. Additionally, atorvastatin treatment suppressed phosphorylation of ERK1/2 and JNK. Furthermore, GGPP, a downstream lipid isoprenoid intermediate, significantly rescued the effect of atorvastatin.

**Conclusions:**

These results suggest that atorvastatin exerts anti-tumoral effects on uterine fibroids through inhibition of cell proliferation and induction of apoptosis in HMG-CoA-dependent pathway. Our results provide the first clinical and preclinical data on the use of atorvastatin as a promising nonsurgical treatment option for uterine fibroids.

## Background

Uterine fibroids are the most common benign tumors in women, with a lifetime incidence of approximately 70% [1, 2]. It is associated with a variety of problems, including menorrhagia, pelvic pain, pelvic pressure, as well as infertility and pregnancy complications [1, 3]. Current therapeutic options mainly involve surgical interventions to remove or destroy uterine fibroids, therefore, uterine fibroids are the major indication for hysterectomy [4, 5]. However, hysterectomy may result into loss of reproductive potential as well as significant morbidity and mortality which are the major limitations of this surgical intervention. To avoid the risks of surgical intervention, many novel therapies are currently under investigation. At present, medical interventions used to manage uterine fibroids include mifepristone [6, 7], ulipristal acetate [8] and GnRH-agonists. Nevertheless, uterine fibroids will re-grow fast after the cessation of mifepristone treatment and the duration of treatment with GnRH-agonists is limited by the induction of hypoestrogenic symptoms [5, 6]. As a result, medical therapeutic options are quite limited for uterine fibroids treatment. Therefore, it is of great clinical value to develop new treatment strategy for uterine fibroids.

Statins are a drug family primarily used for hyperlipidemia. By inhibiting 3-hydroxy-3-methylglutaryl coenzyme A (HMG-CoA) reductase, statins prevent the conversion of HMG-CoA to mevalonate, and thus lead to dramatic reductions in both cholesterol and its isoprenoid precursors; farnesyl pyrophosphate (FPP) and geranylgeranyl pyrophosphate (GGPP) [9, 10]. Statins can inhibit the production of endogenous cholesterol and block protein prenylation, and thus statins use may therefore influence cell proliferation and migration [11, 12]. An increasing evidence suggests that statins use related to several potential anticancer properties and a reduced risk of cancer recurrence, such as hepatocellular cancer, multiple myeloma, breast cancer and ovarian cancer [13–16]. Although statins exhibit multiple therapeutic effects in diverse tumors, limited work has been reported in uterine fibroids. In 2016, Borahay et al. reported that the use of statins was related to a lower risk of uterine fibroids and fibroid-caused symptoms [17]. Simvastatin inhibits proliferation and induces calcium-dependent apoptosis of human uterine fibroids cells in vitro and in vivo [18, 19]. However, whether statins can suppress the growth of human uterine fibroids is not yet well established. Additionally, whether atorvastatin, the most widely used anti-hyperlipidemia in clinic, has an effect on fibroid growth remains unknown. Therefore, in the present study, we proceeded to investigate the antitumor and therapeutic effects of atorvastatin on uterine fibroids as well as its mechanism. We report, for the first time to the best of our knowledge, that atorvastatin treatment exerts anti-neoplastic effects on uterine fibroids through inhibition of cell proliferation and induction of apoptosis in HMG-CoA-dependent pathway.

## Methods

### Ethics statement

This study was conducted according to the principles of the Declaration of Helsinki. It was approved by the ethical committee of the Second Affiliated Hospital of Wenzhou Medical University. A written informed consent was granted from all subjects at the time of enrollment.

### Study population and data collection

Patients with uterine fibroids and hyperlipidemia at the Second Affiliated Hospital of Wenzhou Medical University, Wenzhou, China, between January 1, 2012, and June 30, 2015, were identified. Uterine fibroids were diagnosed by ultrasonography and the diagnosis of hyperlipidemia was followed guidelines published by the American Heart Association [20]. A total of 120 subjects were enrolled with a median age of 44 years (range 34–51 years). All of these 120 cases were intramural myoma (according to evaluation of ultrasonography) and asymptomatic fibroids, in which 53 patients administrated 20 mg atorvastatin everyday, orally (study group), while 67 cases without statins (control group). All individuals were followed up for 2 years.

None of these 120 patients had received medical treatment that might affect the tumor growth of uterine fibroids during the following-up, such as GnRHa, oral contraceptive, estrogen and progesterone drugs or mifepristone. The tumor size in these patients was measured by ultrasonography both before and after atorvastatin treatment. Ultrasound examination of the uterus was performed at 3 monthly intervals. The length (d1), width (d2) and depth (d3) of each tumor were measured, and the volumes were calculated by the following formula: volume (cm^3^) = π × d1 × d2 × d3/6. Volume change was calculated using the following formula: post-treatment volume (cm^3^) − pre-treatment volume (cm^3^).

### Reagents

Atorvastatin, GGPP and FPP were purchased from Sigma Biochemicals (St. Louis, MO, USA). Atorvastatin was dissolved in dimethylsulfoxide (DMSO) and diluted with medium to reach 0, 1, 5, 10, 20, 40 μM before treatment. GGPP and FPP were used at 10 μM.

### Cell line and primary cell cultures

The immortalized human uterine fibroids cell line (HuLM) was a generous gift from William H. Catherino (Uniformed Service University). These HuLM cells were cultured and maintained in DMEM/F12 (Invitrogen, Carlsbad, CA, USA) supplemented with 10% fetal bovine serum and 1% antibiotic (penicillin–streptomycin), in 5% CO_2_ at 37 °C.

Primary human uterine fibroid cells were generated from uterine fibroid specimens collected from the Second Affiliated Hospital of Wenzhou Medical University with an approved protocol. Uterine fibroids tissue samples were collected from 45 Chinese women aged 31–45 years, who underwent hysterectomy to surgically remove confirmed uterine fibroids between Jan 2016 and Jan 2017. The patients were not administered any hormone supplementations including statins for at least 6 months before the hysterectomy was performed. Patients with various complications, such as infections, chronic diseases, uterine malignancy and/or adenomyosis were also excluded.

For preparation of the primary cell population, a portion of the fresh uterine fibroids tissue was washed in cold phosphate-buffered saline (PBS) to remove blood and then chopped into small pieces (1 mm^3^) under sterile conditions, digested with 0.2% (v/v) collagenase II (Invitrogen, Carlsbad, CA, USA) in Dulbecco’s modified Eagle’s medium (DMEM) for 4 h in a 37 °C with shaking. The dissociated cells were centrifuged at 400×*g* for 5 min. The resultant cell deposit was suspended with complete culture medium (DMEM, 10% fetal bovine serum, 100 IU/ml of penicillin G and 100 μg/ml streptomycin) and centrifuged at 400×*g* for 5 min. The resultant cells were cultured at a density of 2 × 10^5^ cells/ml under 5% CO_2_ at 37 °C. Cells from third passages to the seventh were used for the experiments.

### Staining of α-smooth muscle actin by immunocytochemistry

Uterine fibroids cells were identified by the expression of α-smooth muscle actin. Briefly, cells were fixed with 4% paraformaldehyde, permeabilized in PBS containing 0.2% Triton X-100 for 15 min, incubated in a serum-free blocking solution for 15 min at room temperature, and then incubated with mouse monoclonal anti-α-smooth muscle actin antibody (Zhongshan Golden Bridge Biotechnology, Beijing, China) overnight at 4 °C. After extensive washing with PBS, cells were incubated with biotinylated goat anti-mouse IgG as secondary antibody. After incubation the bound antibodies were visualized using 3,3′-diaminobenzidine. Finally, nuclei were stained with hematoxylin. Negative control incubated with PBS instead of primary antibody.

### Cell counting kit-8 (CCK-8) assay

To determine the cell proliferation, 1 × 10^4^ cells/well were seeded into 96-well plates and incubated at 37 °C with 5% CO_2_. After 24 h of incubation, the cells were treated with indicated drugs. By the end of treatments, 10 μl of CCK-8 (Dojindo, Kumamoto, Japan) was added to each well and incubated for an additional 2 h. The reaction product was quantified by spectrophotometry at 450 nm wavelength, and the percentage of viability or number of cells was calculated by formula: (treated cells absorbent/non treated cells absorbent) × 100.

### Flow cytometric assay of apoptosis with Annexin-V FITC Staining

Cell apoptosis was assayed by flow cytometry after Annexin-V FITC staining. Cells were plated at 5 × 10^5^ cells/dish into 60 mm dishes. After reaching 70–80% confluence during exponential growth, cells were harvested, washed with cold PBS and resuspended with binding buffer at a concentration of 2 × 10^6^ cell/ml. Cells were analyzed by using the Apo*Target*™Annexin-V FITC Apoptosis kit (Invitrogen, Grand Island, NY) according to the manufacture’s protocol.

### Western blot analysis

Total proteins were prepared by whole-cell lysis with the buffer (60 mM Tris–HCl, pH 6.8; 5% glycerol; 2% SDS); on ice. The protein from each experimental group was quantified by bicinchoninic acid method (Beyotime, Jiangsu, China). Cellular proteins (30 μg) were solubilized in sample buffer (5× DualColor, ddH_2_O), and heated at 100 °C for 10 min to denature proteins. The proteins were separated by using electrophoresis on 12% sodium dodecyl sulfate–polyacrylamide gel and then electro-transferred onto polyvinylidene fluoride membranes (Millipore, Billerica, MA, USA). The membranes were blocked for 2 h at room temperature in 0.05 M Tris-buffered saline with 0.1% Tween-20 (TBS-T, pH 7.4) containing 5% skimmed milk and then incubated in TBS-T overnight at 4 °C with one of the appropriate primary antibodies at 1:1000 dilutions, unless specified otherwise. The primary antibodies used were PCNA (CST, USA), Bim (CST, USA), Bax (CST, USA), Bcl-2 (CST, USA), caspase 3 (CST, USA), cleaved-caspase 3 (CST, USA), ERK1/2 (CST, USA); p-ERK1/2 (CST, USA); JNK (Abcam, USA), p-JNK (Abcam, USA), α-actin (1:2000; Beyotime, China); and β-tubulin (1:2000; Beyotime, China). After washing with TBS-T, the proteins were incubated with horseradish peroxidase-conjugated secondary antibodies (1:2000 for anti-rabbit-IgG or anti-mouse-IgG) for 1 h at room temperature. Blots then were developed by an enhanced chemiluminescence. The expression levels of above proteins were quantified with densitometry and normalized by corresponding levels of β-tubulin or α-actin respectively. Each experiment was repeated at least three times.

### Human phospho-mitogen-activated protein kinase (MAPK) antibody array

MAPK protein phosphorylation was screened with 300 μg of cell extracts using Human Phospho-MAPK Array Kit according to the manufacturer’s instructions (Proteome Profiler; R&D Systems,minneapolis, MN, USA), which could be used to detect the relative levels of phosphorylation of 26 kinases (Akt1, Akt2, Akt3, Akt pan, CREB, ERK1, ERK2, GSK-3α/β, GSK-3β, HSP27, JNK1, JNK2, JNK3, JNK pan, MKK3, MKK6, SK2, p38α, p38β, p38δ, p38γ, p53, p70 S6 Kinase, RSK1, RSK2, TOR).

### Statistical methods

All statistical analyses were performed with SPSS17.0 software. If each group data was of normal distribution and homogeneity of variance, quantitative data was presented as the mean ± standard deviation while skewed variables were reported as median and interquartile range (IQR). Wilcoxon test or t test was used for comparison as appropriate. Differences among multiple groups were analyzed by one-way ANOVA. Qualitative variables were expressed as proportions and were compared with Chi squared or Fisher exact test as appropriate. A 2-tailed *P* value < 0.05 was considered statistically significant.

## Results

### Atorvastatin suppressed growth of uterine fibroids among patients with uterine fibroids and hyperlipidemia

A total of 120 patients with uterine fibroids and hyperlipidemia were enrolled in this study. Among the 120 patients, 53 patients administrated atorvastatin (study group), while 67 cases without statins (control group). No significant differences were noted in age, body mass index (BMI), tobacco use, parity, and initial tumor size between the study group and control group (*P *> 0.05) (Table 1). The baseline characteristics of the patients were shown in Table 1.

**Table 1:** Baseline characteristics of the patients

Characteristic	Control group (n = 67)	Study group (n = 53)	*P* value
Age (years)	44 (36–51)	45 (34–51)	> 0.05
BMI (kg/m^2^)	22.52 ± 1.94	22.83 ± 1.96	> 0.05
Tobacco use	0 (0)	0 (0)	–
Family history	10 (14.9)	9 (17.0)	> 0.05
Number of gestation	3.0 (1.0–4.0)	2.0 (1.0–3.5)	> 0.05
Number of pregnancy	2.0 (1.0–3.0)	2.0 (1.0–2.5)	> 0.05
Initial volume (cm^3^)	1.86 (0.51–4.82)	1.85 (0.48–4.96)	> 0.05

Data are expressed as mean ± standard deviation, median (interquartile range) or n (%) as appropriate

*BMI* body mass index

**Fig. 1:**
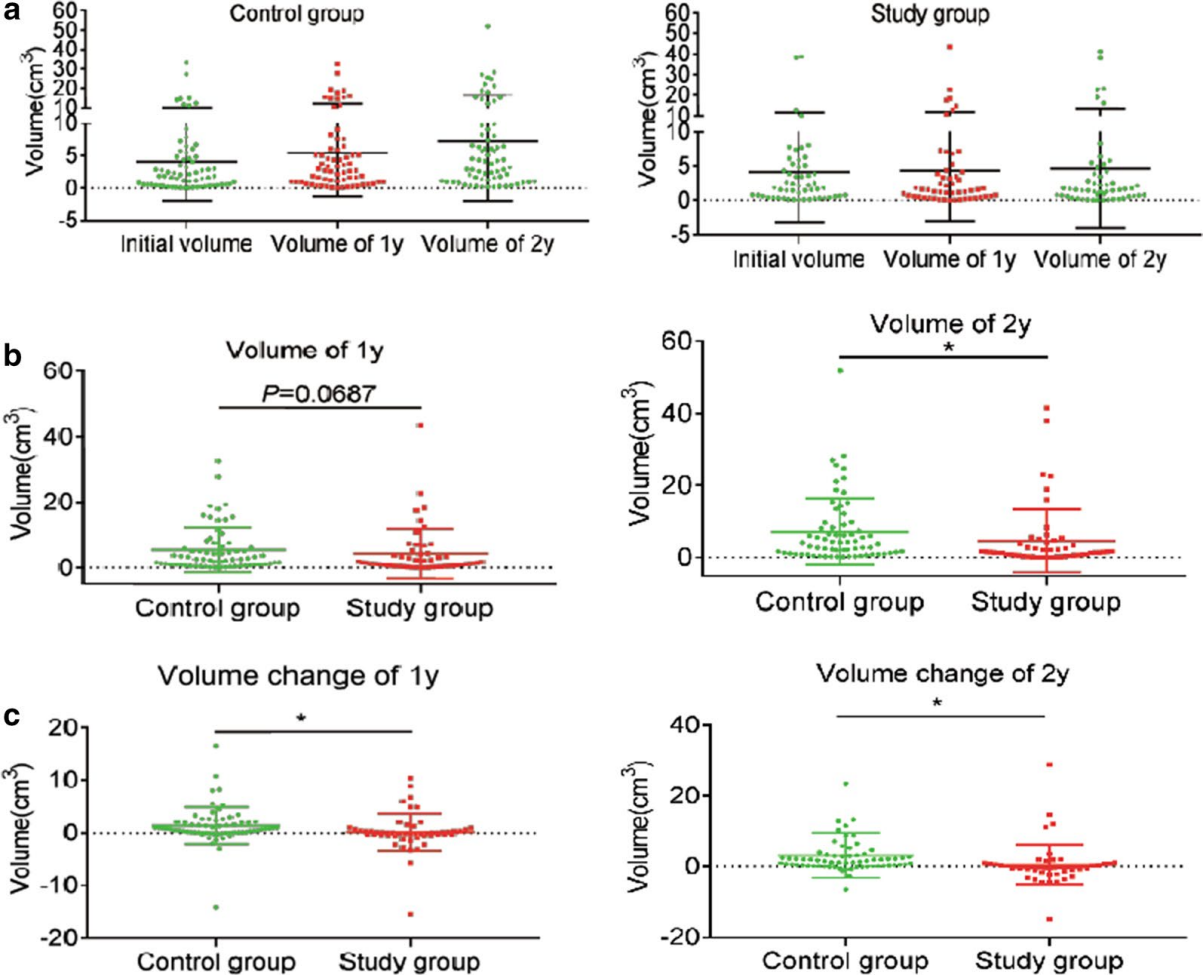
Ever use of atorvastatin and uterine fibroids volume change. **a** Comparative volume of uterine fibroids at initial, 1-year follow-up, and 2-years follow-up; **b** Comparative volume of uterine fibroids between the study group and control group for 1 and 2 years follow-up; **c** Comparative volume change of uterine fibroids between the study group and control group for 1 and 2 years follow-up. Volume change was calculated using the following formula: post-treatment volume (cm^3^) − pre-treatment volume (cm^3^)

Fibroid volume was determined after treated with or without atorvastatin for 1 and 2 years. While the fibroid volume of control group was increased gradually, the study group experienced stable volume (Fig. 1a, b). Totally, after 1 year follow-up, the fibroid volume of 26 cases (49.1%) was decreased in study group, while in the control group, there were only 12 cases (17.9%) presenting reduction volume. After 2 years follow-up, the fibroid volume was decreased in 28 cases (52.8%) of study group and 10 cases (14.9%) of control group. Furthermore, volume change of uterine fibroids was determined after follow-up for 1 or 2 years. As shown in Fig. 1c, volume change of uterine fibroids was much less in study group as compared to control group. Collectively, atorvastatin used for 1 or 2 years significantly suppressed growth of human uterine fibroids.

### Identification of primary human uterine fibroids cells

**Fig. 2:**
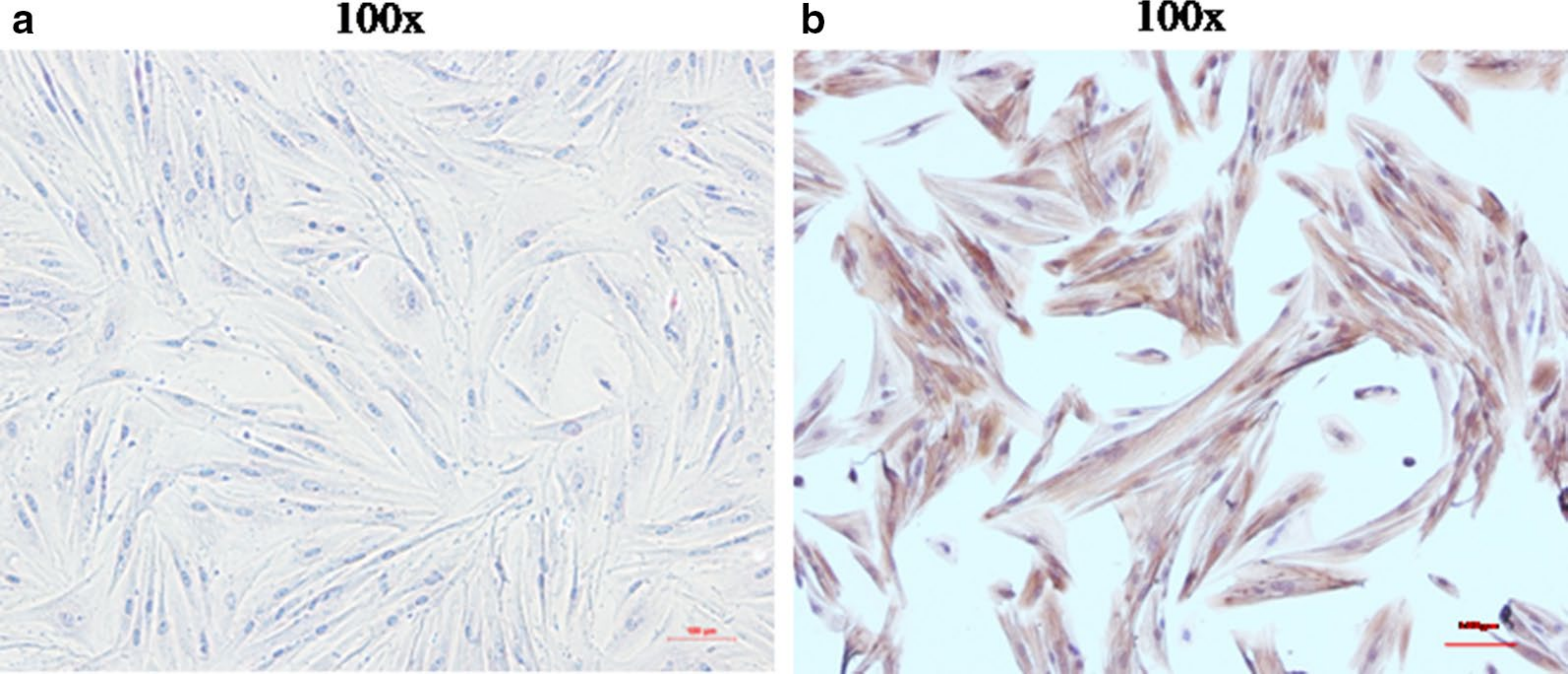
Identification of primary human uterine fibroids cells by immunocytochemistry. **a** Negative control that staining of α-actin in human uterine fibroids cells (SP, ×100); **b** Positive staining of α-actin in primary human uterine fibroids cells (SP, ×100)

To identify primary human uterine fibroids cells (HuLM), immunocytochemistry staining of the cells with α-actin antibody was performed. As shown in Fig. 2, the positive staining of this smooth muscle-specific actin indicated that almost 100% of cells derived from uterine fibroids tissues retained their smooth muscle characteristics.

### Atorvastatin inhibited the proliferation of uterine fibroids cells

**Fig. 3:**
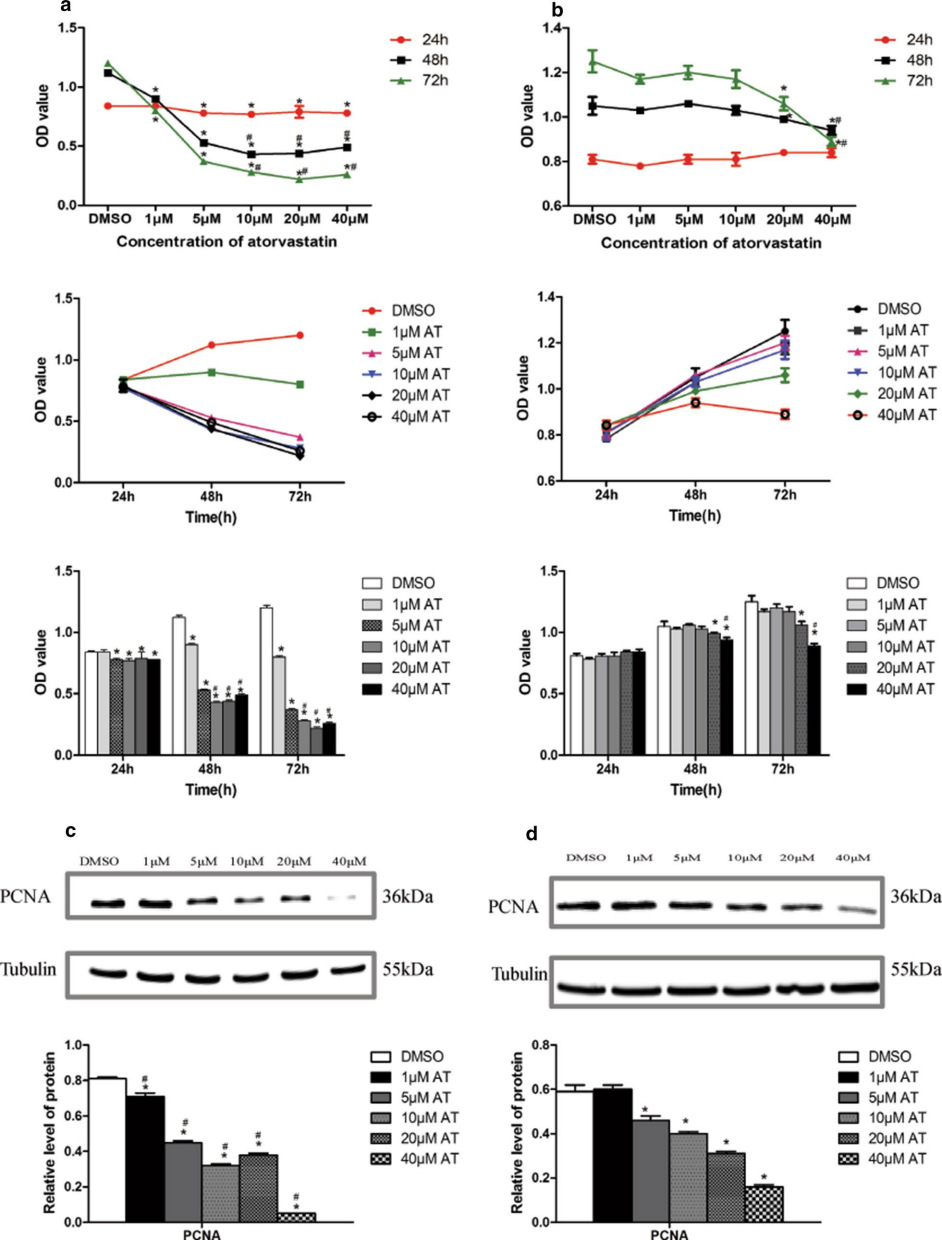
Effect of atorvastatin on cell proliferation in human uterine fibroids cells. Immortalized human uterine fibroids cells (**a**) and primary uterine fibroids cells (**b**) were treated with increasing concentrations of atorvastatin (0, 1, 5, 10, 20 and 40 μM) for 24, 48 or 72 h, and then followed by CCK-8 assay. Data presented were mean ± SEM from triplicate measurements. *P < 0.05 with one-way ANOVA, compared to controls (treated with DMSO). Immortalized human uterine fibroids cells (**c**) and primary uterine fibroids cells (**d**) were treated with increasing concentrations of atorvastatin (0, 1,5, 10, 20 and 40 μM) for 48 h (immortalized cells) or 72 h (primary cells) and then followed by western blot analysis. Tubulin was used as the loading control. The intensity of each protein band was quantified and normalized with corresponding tubulin, and the normalized values were used to generate the graphs. Data presented were mean ± SEM from triplicate measurements. *P < 0.05 with one-way ANOVA

At first, the effects of atorvastatin on proliferation were investigated in vitro by performing dose–response and time-course experiments both in immortalized and primary HuLM cells. Immortalized and primary HuLM cells were treated with various concentrations of atorvastatin (0, 1, 5, 10, 20, 40 μM) for 24, 48 or 72 h, and then followed by CCK-8 assay. In immortalized HuLM cells, atorvastatin significantly inhibited the proliferation in a dose- and time-dependent manner (Fig. 3a). With respect to primary HuLM cells, exposure to atorvastatin for 24 h showed no effect on proliferation, whereas exposure to 20 and 40 μM atorvastatin for 48 or 72 h significantly inhibited the cell proliferation (*P *< 0.05) (Fig. 3b). To further explore the effect of atorvastatin on proliferation, the expression of PCNA was determined by western blot when exposed to different concentrations of atorvastatin. As expected, atorvastatin induced dose-dependent of suppressed the expression of PCNA both in immortalized and primary HuLM cells (Fig. 3c, d).

### Atorvastatin stimulated apoptosis of uterine fibroids cells

**Fig. 4:**
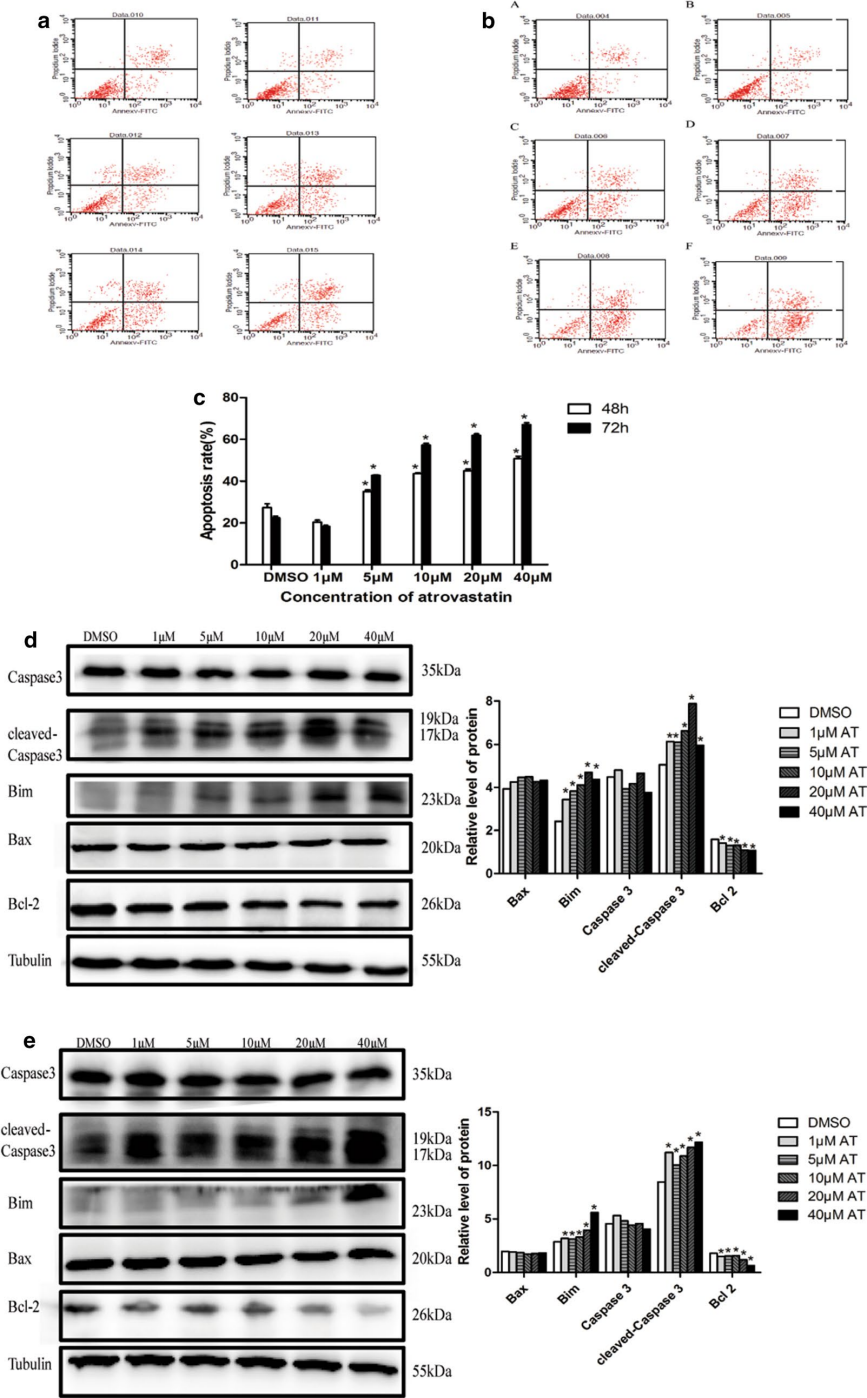
Effect of atorvastatin on apoptosis in human uterine fibroids cells. Immortalized human uterine fibroids cells were treated with increasing concentrations of atorvastatin (0, 1, 5, 10, 20 and 40 μM) for 48 h (**a**) or 72 h (**b**), and then followed by flow cytometry after Annexin-V FITC Staining. Data (apoptosis rate) presented were mean ± SEM from triplicate measurements (**c**). *P < 0.05 with one-way ANOVA. Immortalized human uterine fibroids cells (**d**) and primary uterine fibroids cells (**e**) were treated with increasing concentrations of atorvastatin (0, 1,5, 10, 20 and 40 μM) for 48 h (immortalized cells) or 72 h (primary cells) and then followed by western blot analysis. Tubulin was used as the loading control. The intensity of each protein band was quantified and normalized with corresponding tubulin, and the normalized values were used to generate the graphs. Data presented were mean ± SEM from triplicate measurements. *P < 0.05 with one-way ANOVA

Based on the above observation, to further explore whether atorvastatin-induced antiproliferative effects involved apoptosis, Annexin-V FITC double staining was performed. Again, immortalized HuLM cells were treated with different concentrations of atorvastatin (0, 1, 5, 10, 20, 40 μM) for 48 or 72 h, and then followed by flow cytometry. As shown in Fig. 4, atorvastatin significantly increased immortalized HuLM cell apoptosis compared to control groups (*P *< 0.05) both in 48 h (Fig. 4a) or 72 h (Fig. 4b). To further characterize the molecular mechanisms of atorvastatin-induced apoptosis, apoptosis-associated factors in HuLM cells were determined by western blot upon atorvastatin exposure. As showed in Fig. 4d, e, treatment of immortalized and primary HuLM with atorvastatin up-regulated pro-apoptotic protein cleaved-caspase-3 and Bim as well as down-regulated anti-apoptotic protein Bcl-2 but not Bax. These data demonstrated that atorvastatin induced apoptosis of uterine fibroids cells.

In addition, treatment of immortalized HuLM or primary HuLM with atorvastatin induced dose-dependent morphological changes (data not shown here), which were consistent with the inhibition of cellular proliferation and induction of apoptosis.

### Atorvastatin affected phosphorylation of MAPK pathway

**Fig. 5:**
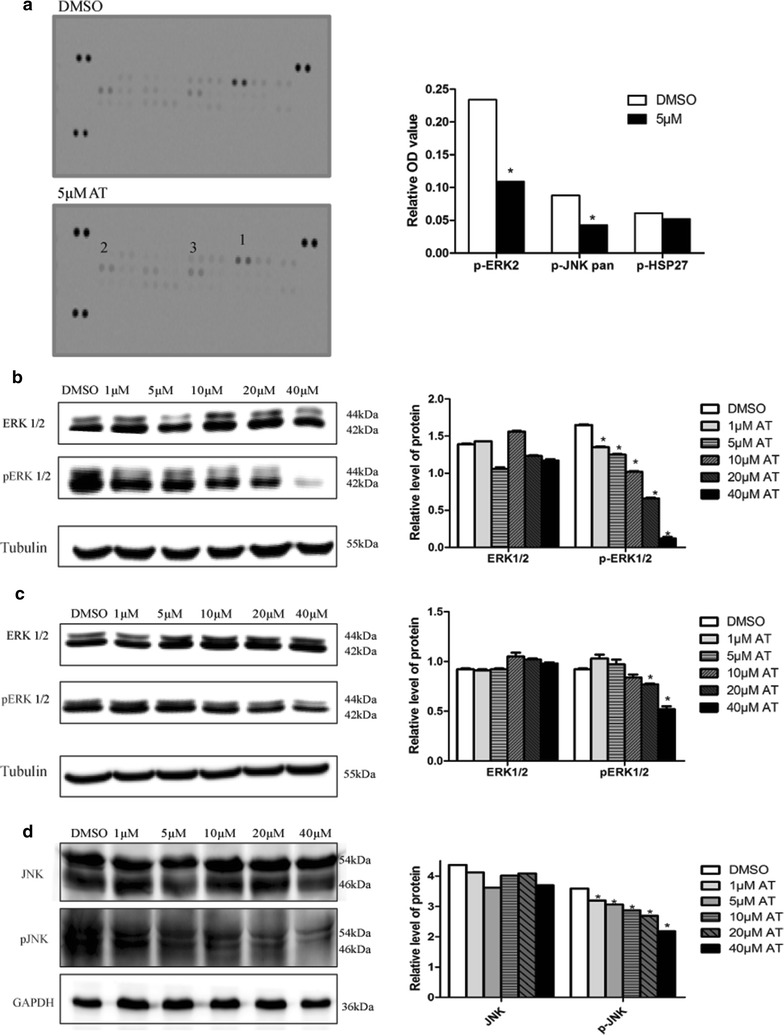
Effect of atorvastatin on MAPK pathway protein in human uterine fibroids cells. **a** Related MAPK pathway protein changed after treated with 5 μM atorvastatin for 48 h in immortalized human uterine fibroids cells (Human Phospho-MAPK Antibody Array). **b**, **c** Effects of atorvastatin on the expression of p-ERK1/2 and ERK1/2 proteins in immortalized cells and primary cells, respectively. **d** Effects of atorvastatin on the expression of p-JNK and JNK proteins in primary cells, respectively. Tubulin was used as the loading control. The intensity of each protein band was quantified and normalized with corresponding tubulin, and the normalized values were used to generate the graphs. Data presented were mean ± SEM from triplicate measurements. *P < 0.05 with one-way ANOVA

To investigate the mechanism(s) by which atorvastatin affects the proliferation and apoptosis of uterine fibroids cells, a standard antibody array technology allowing simultaneous analysis of all three major MAPKs was performed. As shown in Fig. 5a, atorvastatin treatment (5 μM, 48 h) resulted in reductions in the phosphorylation of multiple members of the signaling molecules, including p-ERK2, p-HSP27, and p-JNK pan, while the proteins themselves were not affected significantly, suggesting that atorvastatin affects the signaling pathways mostly by phosphorylation, not by synthesis/degradation of the protein themselves. Western blot analysis essentially confirmed the previous antibody array results, further showing a dose-dependent down-regulation of ERK1/2 phosphorylation both in immortalized and primary HuLM cells (Fig. 5b, c) as well as JNK phosphorylation in primary HuLM cells upon atorvastatin treatment (Fig. 5d).

### GGPP and FPP rescued atorvastatin-associated phenotypes

**Fig. 6:**
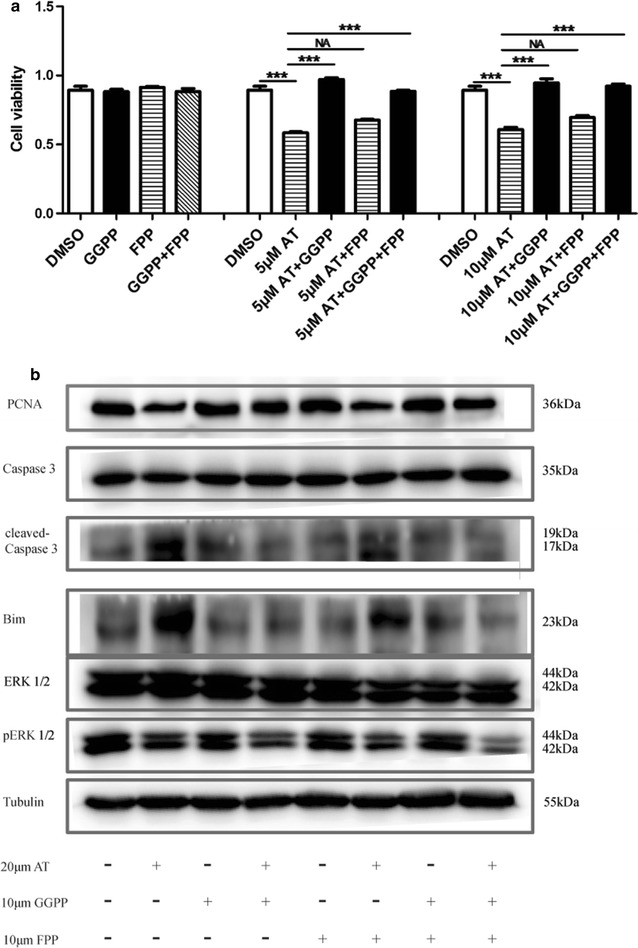
Restoration of atorvastatin-associated phenotypes by GGPP and FPP in human uterine fibroids cells. Immortalized human uterine fibroids cells were treated with atorvastatin (5 μm) with/without 10 μm GGPP/FPP for 48 h, then followed CCK-8 assay (**a**) and western blot analysis (**b**). Tubulin was used as the loading control. Data presented were mean ± SEM from triplicate measurements. *P < 0.05; ***P < 0.001 with one-way ANOVA

Inhibition of HMG-CoA reductase by statins results in the depletion of several intermediates products in the mevalonate pathway. These intermediates included farnesyl pyrophosphate (FPP) and geranylgeranyl pyrophosphate (GGPP)—isoprenoids that served as the substrates for the prenylation of Ras and Rho family of small GTPases, respectively. We next investigated whether supplementation of GGPP and FPP could affect the phenotypes and rescue the effect of atorvastatin in immortalized HuLM cells. As shown in Fig. 6a, GGPP or FPP alone showed no effect on the proliferation of HuLM cells. Notably, concomitant treatment of HuLM cells with GGPP almost completely rescued the cell proliferation induced by atorvastatin, but FPP not (Fig. 6a). Similarly, GGPP or FPP alone showed no effect on the expression of proliferation and apoptosis associated factors, such as PCNA, cleaved caspase 3 and Bim, while GGPP, but not FPP, restored the expression of proliferation and apoptosis associated proteins induced by atorvastatin (Fig. 6b). Interestingly, down-regulated expression of p-ERK1/2 was observed in atorvastatin with the supplementation of GGPP or FPP, and both GGPP and FPP couldn’t restore the expression of p-ERK1/2 induced by atorvastatin.

## Discussion

Statins, HMG-CoA reductase inhibitors and well-known drugs for treatment of dyslipidemia, have been used in clinic for almost 40 years and proved to be safe, effective drugs with minor side effects. Recently, increasing evidences suggested that statins had provocative and unexpected benefits for reducing certain cancer incidence and modifying cancer-related outcomes. Long-term statins use may reduce the risks of glioma [21], colorectal cancer [22], and hepatocellular cancer [13]. Additionally, long-term statins use reduces the mortality of patients with lung cancer [23], prostate cancer [24], and breast cancer [15]. To date, only one study has addressed the risk of uterine fibroids among statins users. In their study, Borahay et al [17]. explored the association between statins use and the risk of uterine fibroids as well as fibroid-related symptoms in a nationally representative sample of commercially insured women diagnosed with hyperlipidemia and reported that the use of statins was associated with a lower risk of uterine fibroids and fibroid-related symptoms. However, the antitumor effects of statins on uterine fibroids have yet to be investigated. There are currently seven statins on the market: atorvastatin, fluvastatin, lovastatin, pitavastatin, pravastatin, rosuvastatin and simvastatin, in which atorvastatin is the most widely used [25]. In the current study, we reported, for the first time, that atorvastatin use could suppress the growth of uterine fibroids by retrospective analysis of 120 patients with uterine fibroids and hyperlipidemia. Our study was based on the observational data, which might have suffered some common limitations of pharmacoepidemiological studies, such as recall bias from self-reported data, detection bias and confounding by indication, as well as selective reporting and other biases. Therefore, further prospective study with large sample size is necessary to confirm these findings.

Atorvastatin reduces serum cholesterol levels mainly by competitively inhibiting HMG-CoA reductase, the rate-limiting enzyme in the mevalonate synthesis pathway [26]. In addition, atorvastatin is highly involved in improving endothelial function, stabilizing plaques, reducing free radical formation and inhibiting endothelial inflammatory reactions, thereby yielding other potential benefits for patients, regardless of cholesterol level reduction [25]. Apart from its well-established therapeutic value in cardiovascular disease, increasing evidences suggest its potential association with cancer. In recent years, a growing body of studies has indicated that statins had potential antitumour effects by inhibition of proliferation, migration and invasion, and induction of apoptosis [25–27]. Our results showed that atorvastatin inhibited the proliferation and stimulated apoptosis of both immortalized and primary human uterine fibroids cells. These results were in accordance with the only previous study exploring the effect of statins on the tumor growth of uterine fibroids. In that report, Borahay et al. demonstrated simvastatin inhibited proliferation, interrupted cell cycle progression, and induced apoptosis through a calcium-dependent mechanism in human uterine fibroids cells in vitro and in vivo [18, 19]. Collectively, atorvastatin can suppress growth of human uterine fibroids through the inhibition of proliferation and induction of apoptosis.

The precise mechanism by which statins suppress the growth of uterine fibroids is not fully understood. By inhibiting the biosynthesis of mevalonate, statins also inhibited the formation of downstream lipid isoprenoid intermediates such as FPP and GGPP [26]. It was proposed that statins could inhibit proliferation in HMG-CoA-dependent [28] or HMG-CoA-independent pathway [29]. Our current study showed that atorvastatin inhibited proliferation and induced apoptosis in human uterine fibroids cells, and these effects could be reversed by adding GGPP, indicating the effects of atorvastatin on uterine fibroids growth depended on HMG-CoA reductase. Interestingly, we found GGPP significantly rescued the effect of atorvastatin, but FPP couldn’t, indicating that blockage of the geranylgeranylation may be more important than prevention of farnesylation for atorvastatin induced human uterine fibroids cells death [30].

Recent evidence showed that statins also could trigger tumor-specific apoptosis in different tumor cells [31]. Statins induced apoptosis in ovarian cancer cells through activation of JNK and enhancement of Bim expression [30]. Both lovastatin and simvastatin induced activation of caspase-8, caspase-3, and caspase-9 in prostate cancer cells [32]. Additionally, lovastatin triggered apoptosis by regulating Raf/MEK/ERK pathway in acute myelogenous leukemia cells [33] and statins activated the mitochondrial pathway of apoptosis in human lymphoblasts and myeloma cells [34]. In the current study, atorvastatin induced caspase-3 activation, up-regulated Bim and down-regulated Bcl-2 in human uterine fibroids cell lines. These results are in agreement with other studies in malignant cells, suggesting atorvastatin triggers uterine fibroids cells to undergo apoptosis via regulating several apoptosis molecules.

ERK1/2 is one of the best studied MAPK signaling pathway members, playing a significant role in the regulation of cell proliferation and apoptosis [35, 36]. Indeed, increased expression of ERK1/2 protein has been detected in uterine fibroids [37] and inhibition of the signaling (MEK1/2-ERK1/2) significantly reduced the proliferation of uterine fibroids cells [37–39]. In the current study, atorvastatin suppressed phosphorylation of ERK1/2 in human uterine fibroids. C-JUN N-terminal kinase (JNK), a member of MAPK family, situated downstream of the small GTPases Ras and Rac1 which regulated a wide spectrum of biological responses such as proliferation, cytokine production, and apoptosis [40]. Meanwhile, high expression of JNK promoted apoptosis significantly in uterine fibroids [41]. In our study, we confirmed that decrease of JNK phosphorylation occurred in uterine fibroids as a result of atorvastatin. These results suggested that atorvastatin affected the human uterine fibroids cells, at least partly, by down-regulating ERK1/2 and JNK activation.

## Conclusions

In summary, atorvastatin notably suppressed growth of human uterine fibroids according to clinical evidences. Atorvastatin can inhibit proliferation and promote apoptosis of human uterine fibroids cells in HMG-CoA-dependent pathway. Mechanistically, these functions of atorvastatin were in part mediated by the activation of ERK1/2 and JNK pathway. Our results provided the first clinical and preclinical data on the use of atorvastatin as a promising nonsurgical treatment option for uterine fibroids. Our future study will further explore the efficacy and safety of atorvastatin for the potential treatment of uterine fibroids using the immune-competent Eker rat model.
